# Rapid Radiations and the Race to Redundancy: An Investigation of the Evolution of Australian Elapid Snake Venoms

**DOI:** 10.3390/toxins8110309

**Published:** 2016-10-26

**Authors:** Timothy N. W. Jackson, Ivan Koludarov, Syed A. Ali, James Dobson, Christina N. Zdenek, Daniel Dashevsky, Bianca op den Brouw, Paul P. Masci, Amanda Nouwens, Peter Josh, Jonathan Goldenberg, Vittoria Cipriani, Chris Hay, Iwan Hendrikx, Nathan Dunstan, Luke Allen, Bryan G. Fry

**Affiliations:** 1 Venom Evolution Lab, School of Biological Sciences, University of Queensland, St Lucia, QLD 4072, Australia; tnwjackson@gmail.com (T.N.W.J.); jcoludar@gmail.com (I.K.); dr.syedabidali@gmail.com (S.A.A.); james.dobson@uqconnect.edu.au (J.D.); christinazdenek@gmail.com (C.N.Z.); danieldashevsky@gmail.com (D.D.); b.opdenbrouw@uq.net.au (B.o.d.B.); jonathan_goldenberg@yahoo.it (J.G.); cipriani.vittoria@gmail.com (V.C.); tropidechis@hotmail.com (C.H.); iwanhx@yahoo.com (I.H.); 2 HEJ Research Institute of Chemistry, International Centre for Chemical and Biological Sciences (ICCBS), University of Karachi, Karachi 75270, Pakistan; 3 Princess Alexandra Hospital, Translational Research Institute, University of Queensland, St Lucia, QLD 4072, Australia; p.masci@uq.edu.au; 4 School of Chemistry and Molecular Biosciences, University of Queensland, St Lucia, QLD 4072, Australia; a.nouwens@uq.edu.au (A.N.); p.josh@uq.edu.au (P.J.); 5 Venom Supplies, Tanunda, South Australia 5352, Australia; nathan@venomsupplies.com (N.D.); luke@venomsupplies.com (L.A.)

**Keywords:** venom, elapid, coagulation, proteomics, evolution, redundancy

## Abstract

Australia is the stronghold of the front-fanged venomous snake family Elapidae. The Australasian elapid snake radiation, which includes approximately 100 terrestrial species in Australia, as well as Melanesian species and all the world’s true sea snakes, may be less than 12 million years old. The incredible phenotypic and ecological diversity of the clade is matched by considerable diversity in venom composition. The clade’s evolutionary youth and dynamic evolution should make it of particular interest to toxinologists, however, the majority of species, which are small, typically inoffensive, and seldom encountered by non-herpetologists, have been almost completely neglected by researchers. The present study investigates the venom composition of 28 species proteomically, revealing several interesting trends in venom composition, and reports, for the first time in elapid snakes, the existence of an ontogenetic shift in the venom composition and activity of brown snakes (*Pseudonaja* sp.). Trends in venom composition are compared to the snakes’ feeding ecology and the paper concludes with an extended discussion of the selection pressures shaping the evolution of snake venom.

## 1. Introduction

The family Elapidae includes some of the world’s most notorious species of venomous snake, including the mambas of Africa, the kraits of Asia, the cobras of Africa and Asia, and the coral snakes of Asia and the Americas. All elapid snakes are venomous, and the fixed hollow fangs situated at the front of the maxilla are one of the family’s principle synapomorphies [1]. A discrete duct connects these fangs to a venom gland associated with compressor musculature, forming a high-pressure venom delivery system that has been likened to a hypodermic syringe [2]. The family is widely distributed and present in many of the world’s tropical and subtropical regions. Throughout most of this distribution, members of the Elapidae compete with both non-front-fanged (NFFs—formerly members of the polyphyletic assemblage Colubridae) and viperid snakes for niche space. In Australia, however, the situation is different: viperid snakes are absent and NFFs are relatively recent arrivals [3]. Consequently, elapid snakes have diversified spectacularly in Australasia, occupying the majority of terrestrial snake niches and even invading the oceans. Australia is home to approximately 100 terrestrial and 30 marine species of elapid snake [4]. Taking into account the fact that all of the world’s sea snakes (Hydrophiinae and Laticaudinae) have Australasian origins [5], the region must certainly be considered the stronghold of the Elapidae.

The broad range of ecotypes present within the Australian elapid snake assemblage provides biologists with a unique opportunity to study the evolutionary relationships between feeding ecology (including prey preference, foraging behaviour and prey-handling behaviour), and venom composition. Unfortunately, few field-based studies have been made of the feeding ecology of Australian elapid snakes. However, due to museum dissection studies, primarily conducted by Rick Shine [3,6,7,8,9,10,11,12,13,14,15,16,17], a substantial amount of baseline data is available regarding the dietary preferences of these snakes. When this data is combined with observations of foraging and prey-handling behaviour, made both in the field and captivity, a basic understanding of the feeding ecology of many species can be achieved.

As has been the case elsewhere in the world, toxinological research of Australian snake venoms has predominantly focussed on large species of snake with well-characterised clinical significance [18]. The majority of Australian elapid snakes, however, are small, cryptic, and seldom encountered by non-herpetologists. In addition, many of them are not considered dangerous to humans and thus have received limited or no research attention from toxinologists. The present study investigates the venom composition of 28 species of Australian elapid snake, several of which have not been previously examined by toxinologists. In addition, the venoms of neonate *Pseudonaja affinis* and *Pseudonaja textilis* are compared to those of their parents, both in terms of composite toxins and clotting activity in human plasma and whole blood (*P. textilis* only). Following a report of the results of these investigations, the paper concludes with an extended discussion of the potential range of selection pressures influencing the evolution of the venom of Australian elapid snakes.

## 2. Results and Discussion

### 2.1. Toxins Detected

In total, 21 classes of toxin were recovered, by “shotgun” MS/MS of crude venoms and MS/MS of gel spots, from the venoms of 28 species of Australian elapid snake (Table 1; supplementary data). Phospholipase A_2_ was the most common toxin class and was detected in the venom of all species except *Vermicella annulata* (27 species in total). It was also undetectable in the venom of neonate *Pseudonaja affinis*, but was present in that of the adult. Three-finger toxins (3FTx) were the next most commonly detected toxin class, being found in all venoms except those of the three species of *Demansia* investigated. Small quantities of 3FTx have previously been detected in the venom of *Demansia vestigiata* [19], however, so it may be that they are also present in the venom of the *Demansia* sp. examined in the present study, but that their low abundance makes them difficult to detect via shotgun analysis. Snake venom metalloprotease (SVMP), C-type lectin (CTL) and the catalytic subunit of the prothrombinase complex (fXa) were also common toxin classes. Consistent with previous studies, the non-catalytic subunit of the prothrombinase complex (fVa) was recovered exclusively from the venoms of *Oxyuranus* and (adult) *Pseudonaja* sp. Other interesting results include the recovery of natriuretic peptides (NP) from the venoms of *Hemiaspis signata* and *Suta punctata* in addition to those of *Oxyuranus* sp., from which they have previously been described, and the preponderance of vascular endothelial growth factor (VEGF) in the venoms of members of the “*Rhinoplocephalus* clade” (*Cryptophis*, *Denisonia*, *Parasuta* and *Suta* [5]). This latter clade is discussed in greater detail below.

**Figure 1:**
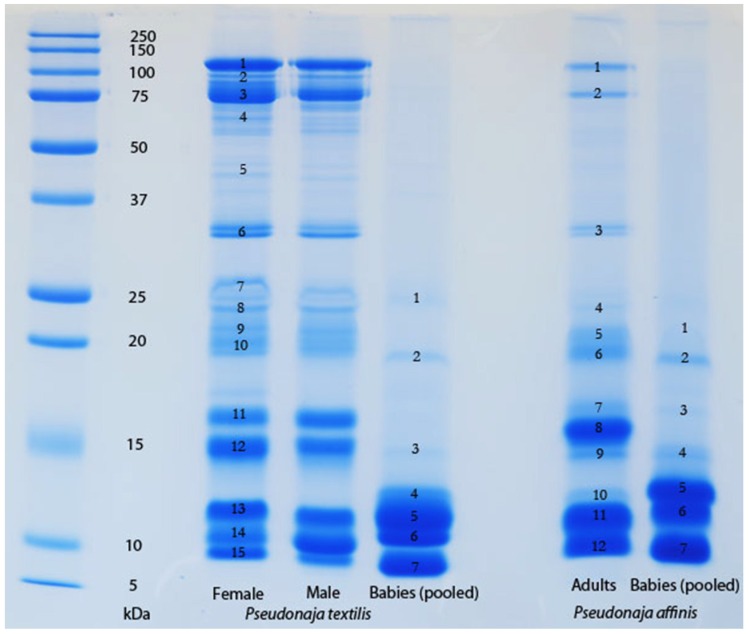
One-dimensional SDS-PAGE comparison of adult (female and male parents) with neonate (pooled) *Pseudonaja textilis* venoms and adult (pooled parents) with neonate (pooled) *P. affinis* venoms. A stark contrast is evident between the venoms of adults and neonates within both species. Particularly noteworthy is the lack of high molecular weight components in the venoms of the neonates, although compositional differences are also evident in the 5–15 kDa range. Toxins identified in adult female *P. textilis* venom (by band number—acronyms are the same as those for Table 1): 1–4 = fVa; 5 and 6 = fXa; 7 = CRiSP; 8–12 = PLA_2_; 13 = NT (no toxin identified); 14 = KP and 3FTx; 15 = 3FTx. Toxins identified in neonate *P. textilis* venom: 1 = CRiSP; 2 = NT; 3 = PLA_2_; 4–7 = 3FTx. Toxins identified in adult *P. affinis* venom: 1 = fVa; 2 = fVa, EN, ADAM, SVMP; 3 = fXa; 4 = 3FTx and KP; 5–8 = PLA_2_; 9 = NT; 10–12 = KP and 3FTx. Toxins identified in neonate *P. affiinis* venom: 1–4 = KP and 3FTx; 6 and 7 = 3FTx.

**Figure 2:**
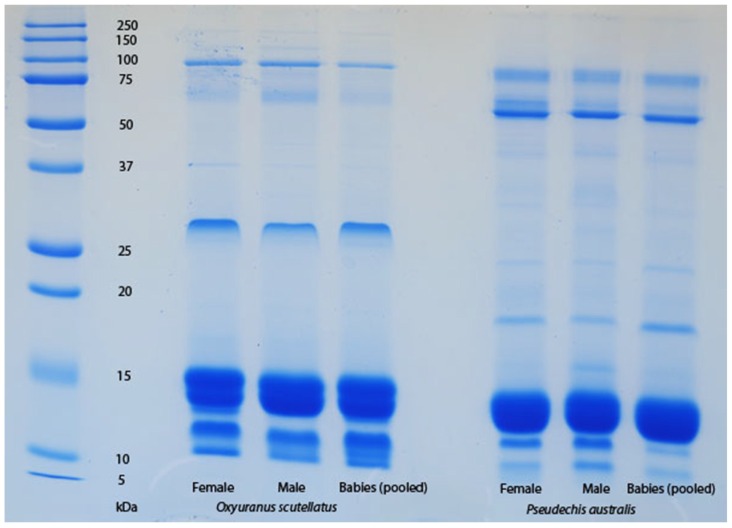
One-dimensional SDS-PAGE comparison of adult (female and male parents) with neonate (pooled) *Oxyuranus scutellatus* venom and adult (female and male parents) with neonate (pooled) *Pseudechis australis*. No ontogenetic shift in venom composition is observable.

**Figure 3:**
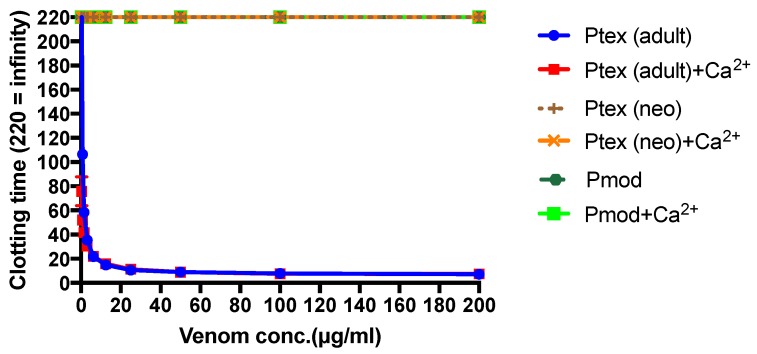
Clotting times following the addition of crude venom to citrated plasma in the presence and absence of calcium. Results for assays in which clots did not form were standardised to 220 s (the longest amount of time after which a clot did form) for graphing. No clots formed following the addition of crude neonate *P. textilis* venom (P. tex (neo)—orange) or adult *P. modesta* (P. mod—green) venom. In contrast, a concentration-dependent clotting effect was observed after the addition of adult *P. textilis* venom (P. tex (adult)—blue). Error bars (where visible) display mean ± SEM.

**Figure 4:**
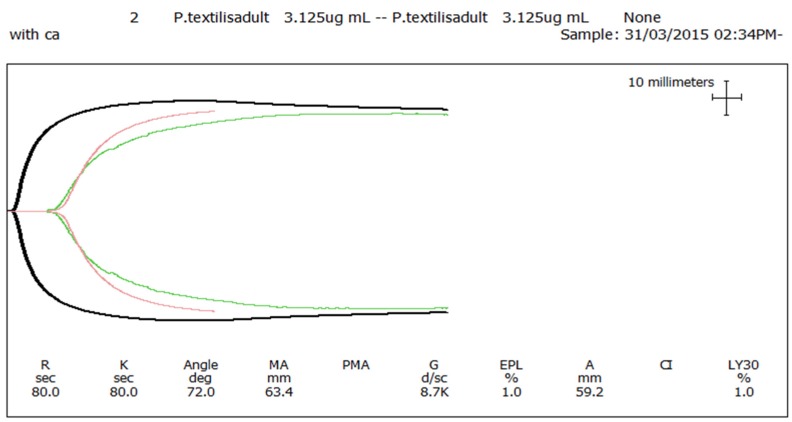
Thromboelastograph comparing clot formation in whole blood following the addition of adult *P. textilis* venom (3.125 μg/mL) + calcium (black), neonate *P. textilis* venom (1 mg/mL) + calcium (green) and calcium alone (pink). Clot formation began almost immediately following the addition of adult *P. textilis* venom + calcium, whereas results forming the addition of neonate *P. textilis* venom + calcium closely match those of the calcium control (the addition of calcium alone, in the absence of venom).

The venom of neonate *Pseudonaja affinis* and *P. textilis* is markedly different from that of adults of the same species (Table 1; Figure 1). In particular, the neonatal venoms are noteworthy for their lack of high molecular weight toxins, including both components of the prothrombinase complex (fVa and fXa), which are present in the adult venoms. A similar shift was not detectable in the venoms of *Oxyuranus scutellatus* or *Pseudechis australis* (Figure 2). The observable shift in the venom composition of *P. textilis* is matched by a shift in the clotting activity of the venom. The venom of neonate *P. textilis* failed to stimulate clot formation in plasma (either in the presence or absence of calcium—Figure 3) or whole blood (thromboelastography—Figure 4) coagulation assays. These results are in stark contrast to those generated for the venom of the adult female *P. textilis* (mother of the neonates) tested in this study, which induced rapid clot formation in both plasma (under both conditions—Figure 3) and whole blood (Figure 4). Consistent with the results of previous studies [18,20], the venom of adult *Pseudonaja modesta* also failed to induce clot formation under any of the tested conditions (Figure 3).

### 2.2. Presence/Absence of Procoagulant Toxins

The catalytic subunit of the prothrombinase complex, fXa, was likely recruited as a toxin near the base of the Australasian elapid snake radiation [18]. It is present in the venoms of both Australian and Melanesian (e.g., *Aspidomorphus muelleri*—Table 1) taxa, but has not been recruited as a toxin by elapid, viperid or non-front-fanged venomous snakes elsewhere in the world. Given that it is an extremely potent toxin, which partially underwrites the extreme toxicity of the venoms of Australian snakes to lab rodents [1,21], its near ubiquity in the venoms of members of this clade is unsurprising (although its detectability may not always translate into a strong procoagulant effect of the crude venom—see [22] and discussion on redundancy below). When combined with the non-catalytic subunit, fVa, it forms an even more devastating weapon—recruitment of the entire prothrombinase complex as a venom toxin is unique to the clade comprised of *Oxyuranus* and *Pseudonaja* [1,5], the most toxic snakes of all [21].

**Figure 5:**
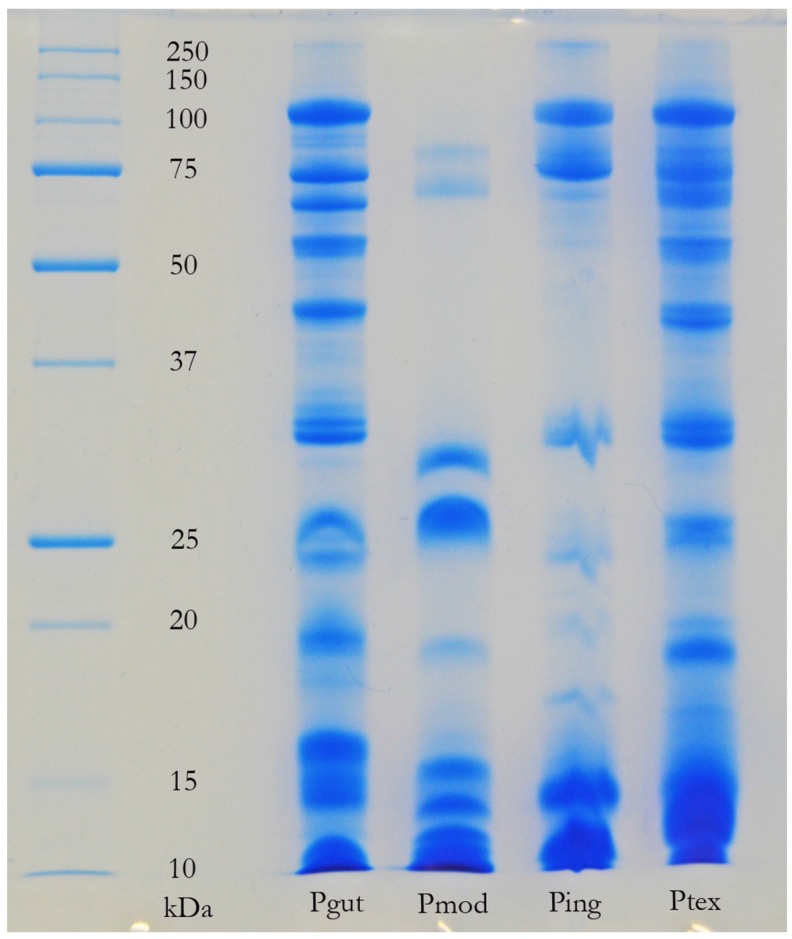
One-dimensional SDS-PAGE comparison of adult *Pseudonaja* venoms illustrating divergence of *P. modesta* from its congeners. Pgut = *P. guttata*; Pmod = *P. modesta*; Ping = *P. ingrami*; Ptex = *P. textilis*.

The absence of detectable quantities of fXa (or serine proteases of any kind) from the venom of certain species of Australian elapid snake investigated in this and previous [18,22] studies is thus equally as interesting as its presence—if a snake is “capable” (genetically) of producing so powerful a weapon, what prevents it from doing so? It is possible that fXa was recruited after the split of *Cacophis, Furina* (and potentially *Loveridgelaps*) from the main stem of the “oxyuranine radiation” [5,18], but this fails to account for its absence from the venoms of *Antaioserpens, Brachyurophis Simoselaps* and *Vermicella* (Table 1). Most striking of all is the absence of the entire prothrombinase complex from the venom of juvenile *P. affinis* and *P. textilis* as well as, apparently, that of adult *P. modesta* (Figure 3 and Figure 5; [18,20]). Although similar ontogenetic shifts have been documented in the venom compositions of certain pitvipers (e.g., *Bothrops jaracara* [23]; *Sistrurus miliarius barbouri*, [24]) this is the first time such a shift has been reported within elapid snakes.

**Figure 6:**
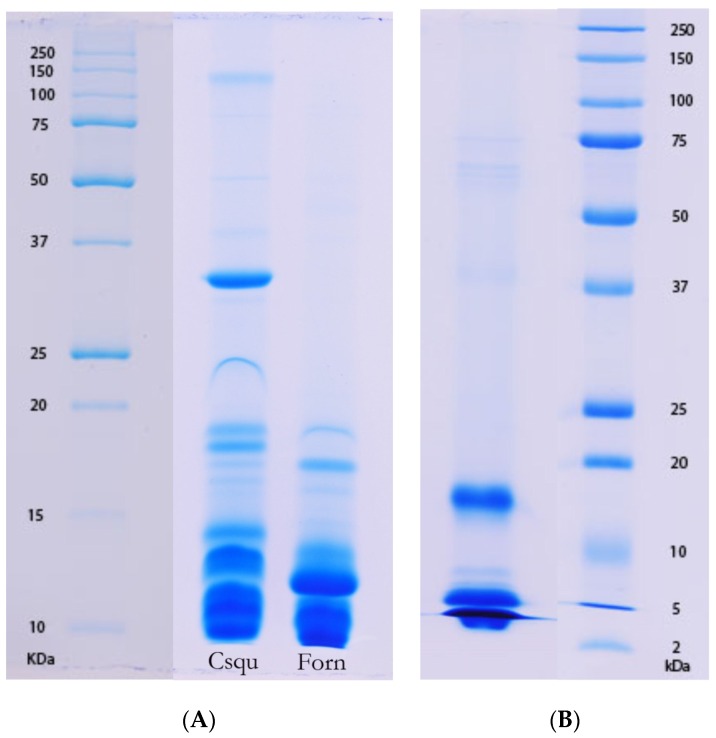
One-dimensional SDS-PAGE of (**A**) *Cacophis squamulosus* (Csqu) and *Furina ornata* (Forn) and (**B**) *Antaioserpens warro* venoms illustrating paucity of high molecular weight content.

When the venom data is compared with the feeding ecology of the snakes, an interesting pattern emerges—almost all the snakes lacking procoagulant toxins (and serine proteases in general) feed on inactive (e.g., dormant) prey. Conversely, almost all those that express and translate these toxins typically feed on active prey. There are exceptions to this trend, of course, in the form of *Acanthophis*, which is unique amongst elapid snakes in being a highly specialised ambush hunter, and the members of the *Rhinoplocephalus* clade, which are notably diverse in both venom composition and feeding ecology (see below) and typically express and translate procoagulant toxins (Table 1; [18]). A further possible exception is *Vermicella*, which is a highly specialised snake that apparently feeds exclusively on blind snakes [7], which may (as both *Vermicella* and its prey are nocturnal) be active when the foraging elapid snake encounters them. Australian snakes that feed on inactive prey are typically nocturnal foragers that feed on diurnal scincid lizards (and/or their eggs), as is the case for *Antaioserpens, Brachyurophis, Cacophis, Furina*, juvenile *Pseudonaja* sp., adult *P. modesta* and *Simoselaps* [3,6,9,15], none of which appear to utilise procoagulant toxins in their venoms (Table 1, Figure 3 and Figure 6; [18]). On the other hand, Australasian elapid snakes that feed on similar species of scincid lizard but catch them during the day, while the lizards are active, e.g., *Aspidomorphus* and *Demansia* (Williams, pers. comm.; [8,17]), include fXa amongst their toxin array (Table 1). It therefore seems plausible that the metabolic state of a prey animal may be equally as influential in determining the toxins appropriate for its subjugation, as the *type* of prey animal (see below).

### 2.3. Complex versus Streamlined Venom Compositions

**Figure 7:**
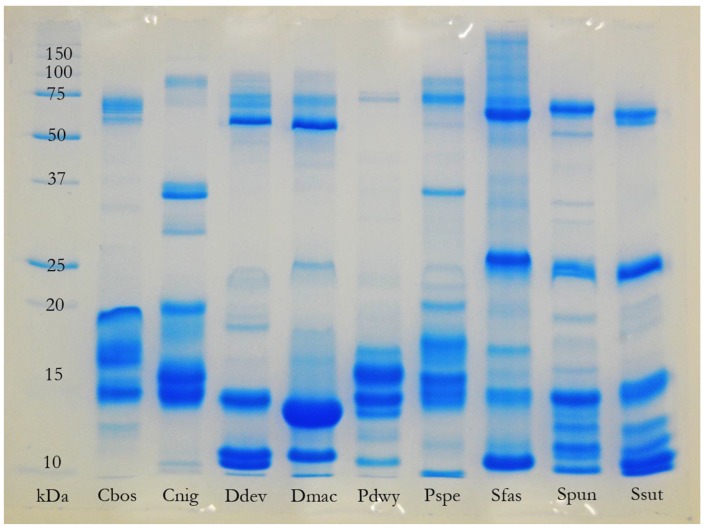
One-dimensional SDS-PAGE comparison of venoms of members of the “*Rhinoplocephalus* clade” illustrating diversity of venom composition. Cbos = *Cryptophis boschmai*; Cnig = *Cryptophis nigriceps*; Ddev = *Denisonia devisi*; Dmac = *Denisonia maculata*; Pdwy = *Parasuta dwyeri*; Pspe = *Parasuta spectabilis*; Sfas = *Suta fasciata*; Spun = *Suta punctata*; Ssut = *Suta suta*.

The most complex venom compositions recovered in the present study, in terms of the number of toxin classes represented, were not those of the “most toxic” species (e.g., *Oxyuranus microlepidotus*), the largest species, or those with the most generalised diets (e.g., adult *P. textilis*). In fact, the greatest diversity of toxin classes was recovered from the venoms of the small-to-medium sized, relatively specialised, members of the genus *Hemiaspis* and the “*Rhinoplocephalus* clade” (particularly *Denisonia devisi*) (Table 1, Figure 7). These results broadly corroborate the results of an earlier study [18] in which *Hemiaspis signata*, *Denisonia devisi*, and *Suta fasciata* exhibited particular diversity amongst toxin classes expressed within their venom gland transcriptomes. What selective pressures drive the evolution of complex, or streamlined, venom composition, if not dietary specialisation? One of a venom’s “trump cards” may be the existence of multiple levels of redundancy within its composition (see below). A “race to redundancy” may drive the evolution of venom towards greater levels of both interclass and intraclass toxin diversity (see below and [25] on the rate of venom evolution in “young” venomous clades). In some lineages, relative streamlining may have occurred following the discovery of certain “good tricks” [26] within specific toxin classes that prevent the evolution of effective resistance in prey organisms without the need for maintaining a high level of intraclass redundancy (see below). This may be the case for the *Oxyuranus/Pseudonaja* clade—following the recruitment of the non-catalytic subunit of the prothrombinase complex, and the subsequent mutation of the entire complex into a devastating exophysiological weapon, the snakes may no longer have experienced selection pressure for the production of a diversity of other toxin classes (however see below for a discussion of interclass functional redundancy in the venom of *Oxyuranus scutellatus*). Similarly, the intraclass redundancy facilitated by the evolution of large numbers of functional isoforms within a single toxin class (e.g., 3FTx in *Acanthophis*, *Pseudonaja modesta* and *Vermicella* [18]) may relieve the pressure to maintain high levels of interclass toxin diversity.

### 2.4. Factors Influencing the Evolution of Australian Elapid Snake Venom

The results of the present study support the hypothesis that a strong link exists, in certain species of snake, between feeding ecology and venom composition. The strongest evidence of this provided here is the demonstration of an ontogenetic shift in the composition and activity of the venom of *Pseudonaja textilis* (Table 1; Figure 1 and Figure 3), a shift in venom that parallels the previously documented ontogenetic shift in the diet of this species [15] and the first time an ontogenetic shift in venom composition has been reported within the Australian Elapidae. That the streamlined venom composition of juvenile *P. textilis* is tailored to their diet of scincid lizards, typically encountered in a dormant state, is corroborated by the fact that a similar venom composition was also observed in other skink and reptile specialists amongst the Australian elapid snakes, such as *Antaioserpens warro, Cacophis squamulosus, Furina ornata*, and *Simoselaps bertholdi* (Table 1; Figure 6 and Figure 7). These results also corroborate those of earlier studies in which ontogenetic shifts in the venom composition of crotalid snakes have been linked to a shift from ectothermic to endothermic prey items [24,27]. The following discussion will consider the possible selection pressures, including those influential during the evolutionary history of a particular species of snake (i.e., that may not be reflected in the present ecological phenotype), that shape the venom composition of Australian elapid snakes.

Venom is a complex, multifunctional system [28,29] and thus the relationship between feeding ecology and venom composition is likely to be similarly complex, with many factors that modify the evolutionary influence of one another. When a venomous snake attempts to secure a meal two complex adaptive systems, predator and prey, enter into a high-stakes interaction, the results of which have consequences of obvious evolutionary importance. A shift in any of the variables that affect firstly the likelihood of an encounter between a particular species of snake and a particular prey organism and secondly the outcome of such an encounter may, in turn, cause a “downstream” shift in other influential variables. The feedback between all contingent variables may therefore have results on venom composition that are difficult to predict. In order to relate feeding ecology to venom composition, it may be instructive to break down “feeding ecology” into a number of somewhat simpler variables: foraging strategy; prey type; prey condition (e.g., the metabolic state of the prey when the snake encounters it); prey-handling (including the influence of the venom delivery system). Although this is not necessarily an exhaustive list of important variables, it is a place to begin. It should be clear that each variable has a strong reciprocal influence on each of the others, such that it will often not be possible to determine a causal hierarchy (i.e., whether particular variables are “upstream” or “downstream” of one another).

### 2.5. Foraging Strategy

Venomous snake taxa vary widely in foraging strategy. One of the major behavioural divisions amongst snakes in general is the way in which they respond to the detection of a scent trail laid down by a potential prey organism. Ambush hunters, such as most viperid, boid and pythonid snakes, conceal themselves next to such trails and simply wait for prey animals (which often use their own scent trails for navigational or territorial purposes). In contrast, active foragers such as most elapid snakes follow scent trails, often tracking prey to their refuges and attacking in close quarters [3].

The hyperdiverse Australasian elapid snake assemblage includes a large number of “typical” (for elapid snakes) active foragers, as well as exceptionally fast snakes that chase down active prey (e.g., *Demansia*); specialised ambush hunters (*Acanthophis*) that combine the focus on scent trails described above with “caudal luring”—wriggling the tail like a worm to attract inquisitive prey animals; species that utilise some combination of the aforementioned strategies; and even “grazers” (sea snakes such as *Aipysurus mosaicus* and *Emydocephalus* that feed upon fish eggs—[30,31]). Naturally, the foraging mode adopted by a particular species has considerable influence on the range of potential prey it is likely to encounter. Specialisation for certain prey types exerts a reciprocal influence on foraging strategy, but it is likely in this case that foraging strategy is more often the “upstream” variable, since a snake must first interact with a particular prey species in order to specialise in feeding upon it.

In addition to overall foraging mode, the period of day during which a snake does the majority of its foraging is likely to substantially influence its feeding ecology. At the simplest level, snakes and their prey items may be divided into nocturnal and diurnal species. Beyond this, it is important to note that a diurnal snake that forages early in the morning may encounter different prey to one that forages in the middle of the afternoon. Similarly, a snake that forages immediately after dark may encounter different prey to one that emerges only several hours after sunset. Furthermore, activity periods of both predator and prey may be extremely plastic, varying according to temperature, rainfall, wind conditions, moon phase. The effects of season and weather on activity periods may co-vary between snakes and their prey, or may vary independently.

### 2.6. Prey Type

Although no experimental investigations of their behaviour have been published, it can be inferred from the reported diets and activity periods of *A. warro, C. squamulosus, F. ornata, P. textilis* and *S. bertholdi* that they are “typical” elapid snakes, i.e., *not* ambush hunters or species that chase down active prey, but active foragers that typically track prey items to their refuges. *A. warro*, *C. squamulosus*, *F. ornata* and *S. bertholdi* appear to be nocturnal foragers throughout their lives [4,6,9]. *P. textilis* often forages nocturnally as a juvenile but transitions towards more diurnal foraging as it increases in size [15]. Diurnal species of scincid lizards feature heavily in the diet of many species of nocturnally foraging Australian elapid snakes, and the five aforementioned species (including juvenile *P. textilis*) are no exception in this regard—*A. warro, F. ornata, S. bertholdi* and juvenile *P. textilis* appear to feed on little else, and scincid lizards make up the bulk of the diet of *C. squamulosus*, although it also takes frogs and reptile eggs. That scincid lizards feature so heavily in the diets of nocturnal Australian elapid snakes can be explained by the extreme diversity and abundance of Scincidae throughout Australia [4], and the fact that the diurnal lizards are sheltering at night, enabling the snakes to follow their scent trails and access them in their refuges.

That activity period has a strong influence on encounters between snakes and prey items is further highlighted by the ontogenetic shift in the diet of *P. textilis* [15]. As mentioned, this species, possibly facilitated by its increasing size and formidable open-mouthed threat display, which combine to reduce the danger it faces from diurnal predators, becomes more active during the day as it increases in size. Other than this shift in activity period, however, the foraging strategy of *P. textilis* appears to remain largely unaltered—it remains a typical elapid snake in this respect, never becoming an ambush hunter or adept at chasing prey down in the open. Diurnally active adult *P. textilis* are opportunistic generalists and will take diurnal prey if given the chance, but their diet differs from its juvenile composition largely in that it includes a higher percentage of nocturnal prey—small mammals. Small nocturnal mammals may be tracked to their daytime refuges using much the same foraging strategy as is appropriate for tracking diurnal lizards at night.

One way that prey type (independent of prey condition) might influence of venom composition is via the relative evolvability of toxins and their targets. In the data presented in the present study a correlation is present between prey class and venom composition. With some exceptions (see discussion on prey condition below), snakes feeding primarily on reptiles (i.e., members of their own taxonomic class) have venoms dominated by low molecular weight peptides, whereas those including large quantities of prey from other taxonomic classes (amphibians and mammals) appear to utilise more enzymatic toxins. The evolutionary rates of proteins are influenced by the robustness of their folding patterns [32], a general observation corroborated by the far greater number of examples of neofunctionalisation, amongst venom toxins, of peptides than globular enzymes [33]. It may be that the chemical arms races driving the evolution of venom composition and resistance exert quite different selection pressures when predator and prey are members of the same taxonomic class and when they are members of different classes. Simply put, it may be “easier” to evolve resistance to enzymatic toxins deployed in intraclass predation because the prey organism’s endogenous inhibitors may recognise the toxins more readily. Indeed, inhibitors of enzymatic PLA_2_ are common in the sera of many snakes, including non-venomous species [34]. This may apply to other enzymatic toxins present in Australian elapid snake venom, although this possibility has not been directly investigated. That Australian lizards are often highly resistant to the venoms of Australian elapid snakes, however, has been demonstrated [35]. Notably, all snakes investigated in that study feed (as adults) primarily on amphibians and/or mammals and have venoms rich in enzymes. Large enzymes are hard to vary without loss of catalytic activity. Although receptors may modify their shape in order to achieve resistance to peptide toxins that would otherwise bind them [36], venoms of snakes favouring peptides often contain large isotypic arrays of these toxins, which may hinder the evolution of resistance in this fashion. This redundancy of targeting (see below) may first arise via the fixation of duplicate genes as the result of selection for increased dosage—the activity of peptide toxins is stoichiometric and thus relatively high concentrations of them are required to inflict a systemic impact on an envenomed prey animal. Redundancy in turn facilitates neofunctionalisation [37,38], so the contingent necessity of increased dosages for peptide toxins may contribute to their evolvability. This conjecture is consistent with the observation that the quantitatively dominant components of certain venoms evolve at a greater rate than components of lower abundance [39].

### 2.7. Prey Condition

As well as influencing prey type itself, a snake’s foraging strategy may also determine the likely “state” that its prey will be in when the snake encounters it. A nocturnal snake feeding on diurnal lizards, for example, will often encounter its prey when the latter is in a dormant or semi-dormant state. As heliothermic ectotherms, the metabolic states of small scincid lizards may vary considerably over the course of 24 hours. The differences between the states of a scincid lizard encountered in its night time refuge and one encountered active during the day may have considerable influence on the suitability of venom and its constituent toxins, as well as prey-handling behaviour, for efficient subjugation. A depressed heart rate or decreased peripheral circulation, for example, may affect the distribution of toxins around the body of an envenomed lizard. Similarly, a decreased metabolic rate may affect the speed at which enzymatic toxins catalyse reactions.

An intriguing observation that suggests a link between prey condition and venom composition can be made by comparing the gross venom compositions of snakes in the genus *Demansia* with that of *F. ornata* (Table 1). These two genera may or may not be closely related (*Demansia* remains something of an enigma within the Australian elapid snake assemblage, being most closely aligned with the exclusively Melanesian genus *Toxicocalamus*), but at the least are similarly early-diverging from the core oxyuranine radiation, which contains the bulk of Australian elapid snake species [5]. *Demansia* sp. and *F. ornata* have similar diets—the latter feeds almost exclusively on scincid lizards, as do the smaller species of *Demansia* (larger species such as *D. papuensis* and *D. vestigiata* may have a slightly more varied diet) [8,9]. Other than prey preference, however, the two genera have strikingly different ecologies—*Demansia* are fast moving diurnal snakes that (at least partially) track their prey visually and are able to chase down active lizards; *F. ornata* is a typical nocturnal elapid snake, utilising scent trails to track lizards to their night-time refuges. These two genera feed on similar prey in dissimilar conditions, and have dissimilar venoms—*Demansia* venoms contain a diversity of high molecular weight enzymatic toxins (Table 1; [19]), whereas F. ornata venom is streamlined and dominated by low molecular weight peptides, similar to that of other elapid snakes (including juvenile *P. textilis*) with similar feeling ecologies (Table 1; Figure 1 and Figure 6). A similar comparison can be made between *Aspidomorphus muelleri* and *Cacophis squamulosus*—both basally divergent members of the Australasian clade [5]. Again, the two species have similar prey preferences (scincid lizards—[6,17]) and in this case both are typical active foragers. A key difference between the two species is that *A. muelleri* is a diurnal forager (Williams, pers. comm.; cf. [17]), whereas *C. squamulosus* is nocturnal [6]. Once again, this difference in foraging period is matched by a difference in venom composition—*A. muelleri*’s venom contains the procoagulant toxin fXa, notably absent from that of *C. squamulosus* (Table 1). This suggests that prey condition may be at least as influential as prey type on venom composition.

It is worth qualifying the fact that our knowledge of Australian elapid snake foraging behaviour is far from comprehensive, as few systematic studies of it have been made. For this reason, speculations such as those above will remain “just so stories” until further investigations are conducted. It may be that such investigations will falsify some of the conjectures put forth in the present paper. This vulnerability to falsification is, in fact, part of the value of reasoned conjecture [40].

### 2.8. Prey-Handling Behaviour and Venom Delivery System

Another variable that is likely to exert (and experience) a reciprocal selective influence on venom composition is prey-handling behaviour. Prey-handling behaviour and venom composition are in turn likely influenced by, and influence, the anatomy of the venom delivery system (including length, position and flexibility of fangs; anatomy of the venom gland; musculature and general anatomy of the jaw and skull). This complex network of influences, even at the relatively specific level of prey-handling behaviour, highlights the importance of taking the “systems perspective” when considering the evolution of venom—individual elements of the venom system do not evolve in isolation from one another.

Several distinct patterns of prey-handling behaviour are evident amongst Australian elapid snakes. Some genera (e.g., *Oxyuranus*) utilise a specialised bite-and-release strategy, some (e.g., large *Pseudechis*) bite and clamp down, maintaining a strong grip with the jaws alone, and many species bite and hold while utilising bodily coils to attempt to maintain a grip on their prey whilst envenoming it. Although this latter behaviour has been referred to as “constriction” (e.g., [41]), it likely differs from true constriction in that the coils are not being used as the primary method of subjugation, but are rather the only “appendages” available to a snake in grappling with prey that may be difficult to envenomate or may take an extended period of time to succumb to the effects of the venom. It is perhaps best considered as part of a “combined arsenal” of prey subjugation strategies. That this form of “grappling” is common in species that feed on scincid lizards may be explained by the osteoderms within the scales of these prey organisms. It may be difficult to maintain a grip on slippery skinks whilst attempting to force a fang in between their scales to effect envenomation. Hence, the widespread utilisation of grappling behaviour amongst Australian elapid snakes may be partially explained by the predominance of these lizards in the diets of many species.

Prey-handling behaviour sometimes varies between closely related genera. *Oxyuranus* and *Pseudonaja* are sister genera, but the former utilises a bite-and-release strategy, whilst the latter often utilises grappling. The bite-and-release strategy of *Oxyuranus* is probably an adaptation for avoiding the risk of retaliation from the dangerous mammalian prey on which this genus specialises [16]. Facilitating this prey-handling behaviour are long, flexible fangs (effective at penetrating thick fur) and the rapid delivery of large quantities of highly toxic venom including the devastatingly potent toxin form of the prothrombinase complex that likely contributes to the extremely rapid collapse of envenomed prey [42]. In contrast, the grappling strategy of *Pseudonaja* is paired with extremely short, stiff fangs, which probably aid in the envenomation of the scincid lizards that are a major part of their diet for much of their lives [15]. The maximum venom yield delivered by *P. textilis* (the largest species of *Pseudonaja*) is also much lower than that of *O. scutellatus* (N. Dunstan, pers. comm.), although the amount of venom delivered during “milking” for venom collection may not be reflective of the amount delivered in a bite to a prey animal under natural circumstances. It may be that *Oxyuranus* typically “meter” their venom, or that peripheral resistance influences the amount injected, as has been demonstrated for some pit vipers [43]. Perhaps they are capable of producing such an abundance of venom because, as mammal specialists, they often have the opportunity to subdue multiple prey animals in a short period of time, e.g., upon discovering a rodent nest. Regardless, prey preferences and the associated prey-handling behaviour have likely influenced the divergence of the venom systems of *Oxyuranus* and *Pseudonaja*, further evidence of which is provided by the lack of any ontogenetic shift in the gross venom composition of *O. scutellatus* (Figure 2).

### 2.9. Prey Defence Mechanisms

A predatory venomous organism must evade or overcome both the external and internal defence mechanisms of its prey. Almost all organisms predated upon by Australian elapid snakes are vertebrates, and the majority of vertebrates attempt to defend themselves by biting, which may, in some cases, pose a serious threat to a predatory snake’s well-being. The bites of mammals such as rodents and small marsupials are particularly dangerous, but some larger scincid (e.g., *Tiliqua*) and agamid (e.g., *Pogona*) lizards, and all varanid lizards, may also be capable of inflicting considerable damage. As discussed above, prey-handling behaviour, such as a bite-and-release strategy, is one way of minimising the danger posed by a retaliatory bite. Adopting this strategy, however, must be facilitated by (and thus exerts a reciprocal selection pressure on) both the venom, in terms of it containing toxins that rapidly incapacitate envenomed prey (thus minimising its escape potential as well as its opportunity to retaliate), and the venom delivery system, which must be capable of rapidly delivering a sufficient dose of venom. These factors may limit the usefulness of such a strategy to certain prey types—mammals have constantly high metabolic rates (facilitating the deployment of fast-acting enzymatic toxins against them) and are not protected by tough skin or osteoderms. A further consideration is the need for some venomous predators to protect themselves from the toxic defences—either venom or poison—of their prey. Amongst Australian elapid snakes, this is particularly relevant to species that feed upon members of their own family (e.g., *Pseudechis*, but also a wide range of other genera). As the evolution of toxin resistance in a venomous predator does not necessarily have an influence on the development of that predator’s own venom system, this interesting variable will not be considered further here.

The predator–prey arms race hypothesis of venom evolution considers overcoming and/or evading a prey animal’s internal defences to be one of the key selection pressures driving the evolution of venom and its constituent toxins [28]. In order to remain an effective strategy for prey subjugation, venom must be able to “stay ahead” of the prey’s capacity to evolve resistance. That venom is so widespread a predatory innovation in the animal kingdom [28] suggests that it is generally capable of achieving this. What properties of venom systems allow them to stay ahead and remain successful strategies? What observable effects do these properties have on venom composition? In order to consider questions such as these, it is again instructive to take the “systems perspective”. The confrontation between a predator’s venom and an envenomed organism’s physiology is a high-stakes battle between two complex adaptive systems, in which strong selection pressures are exerted on both parties: envenomed prey organisms are fighting for their lives and venomous predators are fighting for sustenance. It may seem intuitive that the stakes are higher (and thus the selection pressures stronger) for the prey organism, but if this is so, we must again consider why venoms are so successful.

### 2.10. Gene Duplication and Redundancy

Gene duplication is often considered to be an essentially random process, however, certain factors influence the likelihood of a particular gene being duplicated. These factors include the rate of evolution of the original gene and its contribution to organismal fitness [44]. Subsequent to duplication, the maintenance of additional copies is influenced by both genetic drift and selection. Copies that accumulate synonymous mutations via genetic drift may be maintained, albeit with their expression down-regulated [45], as they do not inflict a fitness cost on the organism. The maintenance of networks of synonymous genes encoding functionally equivalent products is referred to as genetic redundancy. Non-synonymous mutations, on the other hand, are typically either purged by negative (purifying) selection or fixed by positive selection. Deleterious mutations, which compromise the function of the protein encoded by the gene, may be purged, whereas advantageous mutations may facilitate the acquisition of new functions, a process known as neofunctionalisation. An alternate model of duplicate fixation is subfunctionalisation, in which a parent gene with multiple functions, perhaps one primary and one minor or even epiphenomenal, is duplicated and its functions “shared” between daughter genes [46]. This may facilitate selection for the secondary function in one of the copies without compromising the primary function.

A challenge faced by models of gene duplication is to explain the mechanisms that facilitate the maintenance and subsequent evolution of duplicate genes. Duplicates constrained by selection for the original gene’s function are unable to evolve new functions, as purifying selection will quickly purge any non-synonymous mutations. Unconstrained genes, on the other hand, may easily be lost to drift and/or deleted. This challenge is known as “Ohno’s Dilemma” [47]; named in honour of Susumu Ohno, one of the original popularisers of gene duplication as a source of evolutionary novelty [47]. Numerous pathways by which duplicated genes may overcome this challenge have been proposed, but genes encoding venom toxins (and perhaps other secretory proteins) seem particularly suited for doing so. Venom toxin genes are hypothesised to evolve when genes encoding endophysiological regulatory proteins are duplicated and the expression patterns of one of the copies is modified such that it is either recruited or restricted to the venom gland [48,49]. In becoming part of the venom system, the gene’s primary function switches from endophysiological (within the body of the producing organism) to exophysiological (outside the body of the producing organism). This dramatic neofunctionalisation event is what defines a protein as a toxin (perhaps the only way of unequivocally classifying a protein as a toxin is to demonstrate that it has such a function—[50,51]) and simultaneously frees the gene that encodes it from many of the constraints affecting the evolution of gene duplicates that continue to encode endophysiological proteins. The acquisition of an exophysiological function aids toxin genes in decisively escaping Ohno’s Dilemma.

One of the key constraints of endophysiological gene duplicates is dosage-sensitivity. The dosage-balance hypothesis posits that sensitivity to increased quantities of gene products may be a factor in either preventing the duplication of certain genes or the rapid purging of duplicates by purifying selection [45,52]. Thus, in key endophysiological systems, dosage-sensitivity may be a barrier to neofunctionalisation. Conversely, where increased gene dosage is advantageous, the immediate fitness benefit duplication confers may facilitate selection for additional functions or the reification of epiphenomena (fixation of a duplicate by selection for a previously unselected property of the gene product) [53]. Venom genes escape the deleterious effects of dosage-sensitivity via their selective amplification in the venom gland. Indeed, increased dosage of venom toxins, particularly those with stoichiometric activities (e.g., non-enzymatic peptides), is likely to confer a fitness benefit to the venomous organism by increasing the overall toxicity of the venom and facilitating rapid replenishment of venom stocks after glandular depletion [54]. The duplication of venom genes, therefore, may be far less constrained than that of genes encoding regulatory products, a hypothesis supported by the unusual frequency of duplication amongst certain venom peptides [55].

A consequence of relaxed constraints on the maintenance of duplicate copies in venom systems is a higher degree of genetic redundancy than that observed in typical endophysiological systems, likely including those targeted by the toxins. This property of venom systems may enable them to “out-evolve” the molecular defences of prey organisms. Redundancy is a common property of physiological systems involved in the maintenance of homeostasis [56]. Large numbers of paralogs (gene duplicates) often encode proteins with the same function [57]. This ensures that a copy affected by a deleterious mutation can be purged by purifying selection, whilst other copies remain unaffected and the overall function of the system of which they are an integral part remains unperturbed—in general, redundancy provides stability [58]. Mutations are not always deleterious, however, so if a mutation enables a paralog to acquire a potentially beneficial new function, this function can be positively selected because other paralogs remain unchanged and continue to provide stability—redundancy is important for neofunctionalisation [37,38]. In order to maintain redundancy, however, duplicate genes in systems vulnerable to dosage-sensitivity must have their expression levels tightly regulated: the more synonymous copies the lower the expression level (i.e., the contribution to the overall dosage) of each copy must be [45]. Venom genes, however, can remain individually highly expressed even when part of massively redundant networks. This may facilitate a higher rate of evolution amongst the dominant components of a venom [39], as advantageous mutations are more likely to be “seen” by selective forces. This idea is corroborated by the dynamic evolution observed in certain venom toxins [55,59]. If venom toxins attack systems unable to match the degree of genetic redundancy they exhibit, this may contribute to giving venomous predators the evolutionary “edge” in predator–prey arms race scenarios.

Unlike endophysiological systems, venoms, systems with exophysiological functions, exhibit two levels of redundancy. These two levels may be described as redundancy at the level of the target and redundancy of function at the systems-level. Redundancy at the level of the target is the equivalent of genetic redundancy, except that the “target” in an endophysiological system is whatever the gene product in question interacts with, whereas the target of a venom toxin is within a physiological system of another organism. Redundancy of targeting exists when two or more toxins (often isoforms of the same toxin class) share the same target at the molecular level. In Australian elapid snake venoms, this form of redundancy is easily observable in alpha-neurotoxins—the venom of a single species of snake may contain numerous isoforms of 3FTx (46 distinct isoforms of 3FTx were recovered from the venom gland transcriptome of *P. modesta*—[18]), which may target the same postsynaptic nicotinic acetylcholine receptors (nAChr—although 3FTx isoforms may differ in their affinity for different subtypes, an example of subfunctionalisation). Redundancy of function at the systems-level occurs when two or more toxin classes (or sub-systems of toxins—see discussion of toxin synergy below) exist in the same venom that are each capable of independently fulfilling the primary function of the venom. For example, the venom of *O. scutellatus* contains an exceptionally potent procoagulant enzyme complex that disrupts haemostasis in envenomed prey animals and rapidly incapacitates them through stroke-induction [42]. The same venom includes slower acting, but equally lethal, toxins—a pre synaptic neurotoxin complex (taipoxin, a heterotrimeric PLA_2_—[60]) and a calcium-channel blocker (taicatoxin—composed of an alpha-neurotoxin, a PLA_2_ and a kunitz-type serine protease inhibitor—[61]). Any one of these toxins is lethal to rodents in isolation and thus potentially capable of fulfilling the primary function of the venom—prey subjugation.

That these multiple levels of redundancy exist within venoms is likely of considerable evolutionary importance and toxinologists pondering the evolutionary “justification” of the venom compositions they observe may benefit from considering the role of redundancy in shaping them. The importance of extra levels of redundancy in venom systems may lie in their giving venom the “edge” in predator–prey arms races (e.g., Northern Pacific rattlesnakes outstripping their California ground squirrel prey [62]. Having numerous toxin isotypes within the same venom that target the same receptor may, as well as increasing the possibility of toxin neofunctionalisation, decrease the likelihood of resistant receptors evolving, because any novel mutation that confers resistance to one isotype may not confer resistance to another. Such a potentially beneficial mutation may thus be prevented from going to fixation within a population because it may not provide a selectable benefit in terms of overall venom resistance. Redundancy of function at the systems-level may be an even more powerful evolutionary “trump card”—even if resistance to one toxin type arises, this cannot be selected for if another toxin type kills the prey in any event. For example, if a mutation arose in a rodent that enabled the production of a prothrombinase-inhibitor with selective affinity for the procoagulant complex in *O. scutellatus* venom, such an innovation might not be selected for unless the rodent simultaneously developed resistance to both taipoxin and taicatoxin as well. This fact may go some way towards answering the question of why some venomous organisms have venom that seems to be far more potent than it “needs to be”—this extreme potency may be partly the result of the functional redundancy that exists to thwart the evolution of resistance.

It is worth clarifying, in light of the above discussion, that gene duplication itself need not be adaptive for redundancies such as those described to arise. Duplication itself may be contingent, but the fact that a gene is present at a locus that is subject to higher levels of duplication than average (e.g., one without pleiotropic constraints—[44]) may in fact contribute to its co-option as a venom toxin. Regardless, selection acts on the fixation or purification of duplicate genes after the fact. We have discussed above ways in which venom toxins may escape the influence of purifying selection. In addition, selection for increased dosage is likely to fix duplicate venom genes as this may increase the toxicity of the venom. This is particularly salient for toxins with stoichiometric activities such as peptides, which indeed exhibit the highest duplication rates [55]. The influence of evolutionary history is also important—past arms races may have contributed to the development of redundancies as outlined above, so a venomous organism need not experience strong selection (via the evolution of resistance in prey animals) during its own lifetime in order to maintain the massively redundant toxin arsenal it inherited from its ancestors, it need only not be penalised for it. The development of redundancy, like all things naturally selected, must begin with contingencies—evolution by natural selection is, after all, the reification of past contingency. Furthermore, it is implicit within the venom redundancy hypothesis sketched in the present paper that not all toxins detectable within a given venom necessarily *function* as toxins within the present venom phenotype. They may have been important toxins in the past, which are now expressed in low, vestigial, quantities (pending a change of selective regime which may see them once again called into action) or they may simply be members of gene families with appropriate expression profiles that have not previously been utilised as venom toxins within the present lineage. Discussion of these potential variants of adaptive and non-adaptive (epiphenomenal or “exapted”) redundancy is beyond the scope of the present paper, but is the subject of an upcoming theoretical study.

### 2.11. Toxin Synergy and Negative Selection

That multiple toxins in a single venom may interact, one facilitating or potentially aiding the effect of another, is well documented [63]. A classic example of this is toxin complexes in which multiple toxins are bound together—sometimes the components are individually toxic, sometimes completely non-toxic, but generally the toxicity of the whole complex far exceeds that of any component in isolation. Synergistic toxins are not always bound together in complexes—in elapid snake venom it is not uncommon for both pre- and post-synaptic neurotoxins to be present, the combined effect of which is likely a more comprehensive neurotoxic impact on prey organisms. There are many such examples of positive synergy in venoms, but less consideration has been given to the possibility of negative or competitive synergy, in which one toxin interferes with the action of another.

If a complex venom composition results in functional redundancy and thus helps prevent prey organisms evolving resistance, why do some venomous organisms appear to have far simpler venom compositions than they are (genetically) capable of producing? This is the case with the venom of juvenile *P. textilis*, as demonstrated in the present study, and also that of *P. modesta*, which, as a member of the genus *Pseudonaja,* is descended from an ancestor (the MRCA of *Pseudonaja* and *Oxyuranus*) that expressed the venom gland-specific toxin form of the prothrombinase complex. These snakes have the ability to produce a devastating procoagulant toxin (although *P. modesta* apparently no longer even expresses the fXa gene [18]), which is perhaps the primary weapon in the venom of adult *P. textilis*, particularly in specimens from Queensland [64]. That juvenile Queensland *P. textilis* do not produce this toxin suggests that there is some negative selection pressure that precludes its production during this life stage. It may be the case that the procoagulant is simply ineffective at subduing the lizards (typically encountered in a dormant state—see above) that are the primary food of juvenile *P. textilis*. As making the enzyme complex (which consists of two large molecules) in appreciable quantities presumably incurs a metabolic cost, the translation of the gene that encodes it may be blocked for this reason. This metabolic cost may not be significant [65], however, and thus may not be sufficient to prevent the enzyme’s production (although see [54] for further discussion). Alternatively, it may be the case that the presence of this toxin in the snake’s venom would not only not aid in the subjugation of dormant scincid lizards, but, in addition, would actually hinder it.

There are at least two ways in which the presence of an enzymatic procoagulant toxin in venom might hinder the subjugation of dormant scincid lizards: production of the enzyme might lower the concentration of important peptide toxins beneath a critical efficacy threshold, and/or the activity of the enzyme in the prey animal might hinder the delivery of peptide toxins to their binding sites. Venom yields are limited by the development of the venom gland and its associated anatomy (including musculature, fangs, ducts). *Pseudonaja* are typically low yielders in comparison to similarly sized *Pseudechis* or *Oxyuranus*, (although particularly large specimens may yield a considerable amount of venom [66]), and the venom yields of juvenile *P. textilis* are tiny (TNWJ pers. obs.). Given this fact, devoting a percentage of the total yield to an ineffective toxin might considerably hamper the efficacy of the venom as a whole. As previously discussed, the dormant scincid lizards on which juvenile *P. textilis* feed likely have a depressed heart rate and overall metabolic state, although no experimental validation of this assertion exists. If indeed this is the case, as well as limiting the efficacy of enzymatic toxins, a depressed heart rate and peripheral circulation may lead to high concentrations of toxins building up at the bite site. High concentrations of procoagulant toxins would likely result in the formation of large clots localised around the site of envenomation, which may prevent the systemic delivery of important peptide toxins. One physiological defence against envenomation is to attempt to trap toxins near the bite site by deploying neutrophils to generate an extracellular trap [67]. The formation of a large thrombus at the bite site may have a similar effect, in this case resulting in an envenomation that fails to subdue the intended prey item. If this is the case, it is an example of negative synergy in which toxins do not work together but work against one another. The interference of the procoagulant toxin, in preventing the systemic delivery of peptide toxins, may thus constitute a strong negative selection pressure that results in the exclusion of the former from the venom despite the decrease in functional redundancy this entails.

### 2.12. Evolutionary History

Evolutionary biologists are constantly mindful of the fact that the study of extant organisms provides us with a snapshot from which we (in conjunction with bioinformatics and palaeontology) may attempt to infer the details of past selection regimes. When interpreting the evolution of venom composition, it is important to note that the present state that we can observe directly is contingent on past states that we cannot. Thus, the presence or absence of certain venom components may not always be adaptive. The common ancestor of all snakes may have been a nocturnal forager that fed on slender prey items that were tracked by following scent trails and attacked in confined spaces [68]. This ecology is similar to that of many small Australian elapid snakes and may indeed be the plesiotypic state for the Elapidae as well as that of snakes in general. Although large and conspicuous snakes like cobras (*Naja*), mambas (*Dendroaspis*) and taipans (*Oxyuranus*) may leap to mind as archetypal elapid snakes, these genera are likely highly derived. The basally divergent coral snake radiation (of the Americas and Asia) may be closer to the ancestral state and small nocturnal foragers occur everywhere the family does. Amongst the Australian assemblage, some of the representatives of this putatively ancestral ecotype exhibit a streamlined venom composition, composed primarily of low molecular weight peptides, but others do not.

The *Rhinoplocephalus* clade is highly diverse ecologically, but many of its members (e.g., those from the genera *Cryptophis, Parasuta* and *Suta*) are nocturnal foragers that feed primarily on scincid or agamid lizards [11,14]. Unlike many of the other genera that occupy this niche (discussed above), the venoms of these snakes, as well as others in the *Rhinoplocephalus* clade, appear notably diverse (Figure 7). That the ecology of the clade is also diverse and includes, in addition to typical nocturnal foragers, diurnal foragers (*Elapognathus*), nocturnal ambush-hunting frog specialists (*Denisonia*), and a true generalist (*Suta suta*), suggests that the entire clade may be descended from a generalist. If this is indeed the case, the nocturnal-foraging lizard specialists may be atavistic in their ecology, and the complexity of their venom (which lacks an equivalently potent procoagulant toxin to that present in the venom of adult *P. textilis*) may be preserved in order to maintain functional redundancy. On the other hand, reptile specialists with streamlined venom may (with the obvious exception of juvenile *P. textilis*) come from a long line of reptile specialists, in which selection pressures never favoured the recruitment of additional toxins into the venom arsenal. Regardless, the venom and evolutionary relationships of the fascinating *Rhinoplocephalus* clade, which also includes species capable of inflicting potentially life-threatening bites to humans (e.g., *Suta suta*), deserve far more research attention than they have received to date.

## 3. Conclusions: The “Race to Redundancy” and Speculations on the Evolutionary Trends Observable in the Venom Compositions of Australian Elapid Snakes

Another possible explanation for the diversity of venom components present in the venoms of members of the *Rhinoplocephalus* clade, and indeed for many of the observable trends in venom composition (and overall phenotype) throughout the whole Australasian elapid snake radiation, may be the “youth” of the clade. The entire radiation, including some 120 terrestrial (between Australia and New Guinea) and more than 60 marine species, may be less than 12 million years old and is not more than 25 million years old [5]. Within an evolutionary blink of an eye, the clade has diversified to occupy a wide range of terrestrial habitats as well as invading the ocean. The reason for this is clear—when elapid snakes first arrived in Australia there were very few snakes here. Indeed, the entire non-front fanged advanced snake (Caenophidia) radiation, that occupies a majority of terrestrial snake niches elsewhere in the world, was absent. As the clade is so young and has diversified so rapidly it is unsurprising that the evolution of the venom of its members should be similarly dynamic. Whereas in other parts of the world and in other venomous clades (particularly the ancient clades of venomous invertebrate) we may only be able to see the end results of extended periods of “evolutionary tinkering”, in a clade as young as the Australian Elapidae we may be able to see the *tinkering itself* represented amongst extant species. This may in fact be the case for the reptilian venom system in general [1], but the Australian Elapidae are a particularly young and particularly dynamic clade even amongst venomous squamates.

A hypothesis, supported by bioinformatic evidence, has recently been proposed—that venom systems exhibit a “dual speed” evolutionary profile [25]. In young venomous clades, toxins evolve rapidly under the influence of positive selection, whereas in ancient clades they are typically constrained by negative selection. Taking the systems perspective, this could be interpreted as a “race to redundancy”—once sufficient levels of redundancy have been reached, and enough “good tricks” [27] have been discovered, the evolutionary brakes are applied and venoms purified, focussed around the most devastating weapons discovered during the initial rapid phase. The idea that venoms may pass through complexity thresholds before purification, in the form of gene deletions, streamlines them is supported by a recent study of the evolution of PLA_2_ in rattlesnakes [69]. The authors of that study came to the conclusion that the MRCA of rattlesnakes possessed up to seven venom PLA_2_ genes, whereas the extant species examined had between three and five. If this complexity-to-purification trajectory is a widespread phenomenon in the evolution of venoms, and if we are able to observe this taking place amongst the extant Australian elapid snakes, then the interclass complexity of the venoms of (e.g.,) *Hemiaspis* and members of the *Rhinoplocephalus* clade might represent a peak in the race towards interclass functional redundancy. The more “refined” venoms of (e.g.,) *Oxyuranus* and *Pseudonaja*, on the other hand, may have already crossed that threshold—enough “good tricks” having been discovered, they have entered a period of “consolidation”. The same might be said for each of the clades of large, conspicuous Australian elapid snake (e.g., the *Notechis* clade, *Pseudechis, Acanthophis*—each has passed through a complexity threshold (a “critical point”) and reached a level of specialisation reflected in their derived phenotypes: body size; prey-handling strategy; venom-delivery system and venom composition.

The majority of Australian elapid snake species remain almost completely neglected by toxinologists and indeed by biologists in general. The data presented in the present article barely scratches the surface, but is enough to begin evaluating the existence and character of trends influencing the evolution of venom within this spectacularly diverse clade. As toxinologists continue to work on the clade, further elucidating the composition and activities of the venoms of its members and relating these data to what is currently understood of their ecology and evolutionary history, it is to be hoped that the attention of evolutionary biologists from a diverse range of subdisciplines will be captured. Venom is a complex adaptive system (CAS) that may eventually be acknowledged as a perfect model for the study of CAS in general. Amongst venomous clades, few are likely to exhibit a more dynamic recent evolutionary past than the Australo-Papuan elapid snake radiation, making these snakes particularly, and perhaps uniquely, attractive in this regard. It is hoped that this preliminary paper helps galvanise future research in this fascinating area.

## 4. Materials and Methods

### 4.1. Venoms

Venoms were sourced either from the long-term research collection of the Venom Evolution Laboratory, or donated by Nathan Dunstan of Venom Supplies Pty Ltd. Venoms were milked from wild caught specimens except where specified. Species abbreviations: Aan—*Acanthophis antarcticus*; Amu—*Aspidomorphus muelleri*; Awa—*Antaioserpens warro*; Cbo—*Cryptophis boschmai*; Cni—*Cryptophis nigrescens*; Csq—*Cacophis squamulosus*; Dde—*Denisonia devisi*; Dpa—*Demansia papuensis*; Dps—*Demansia psammophis*; Dri—*Demansia rimicola*; For—*Furina ornata*; Hbi—*Hoplocephalus bitorquatus*; Hbu—*Hoplocephalus bungaroides*; Hst—*Hoplocephalus stephensii*; Had—*Hemiaspis damelii*; Hsi—*Hemiaspis signata*; Lel—*Loveridgelaps elapoides*; Osc—*Oxyuranus scutellatus*; Omi—*Oxyuranus microlepidotus*; Pdw—*Parasuta dwyeri*; Psp—*Parasuta spectabilis*; Paf(a)—*Pseudonaja affinis* (adult); Paf(b)—*Pseudonaja affinis* (neonate); Pte(a)—*Pseudonaja textilis* (adult); Pte(b)—*Pseudonaja textilis* (neonate); Sbe—*Simoselaps bertholdi*; Sfa—*Suta fasciata*; Spu—*Suta punctata*; Ssu—*Suta suta*; Van—*Vermicella annulata*.

### 4.2. 1D Gels

The 1D gel method utilised the following specific conditions: 1 mm 12% SDS-Page gels with resolving gel layer (3.3 mL Milli-Q H2O, 4 mL 30% acrylamide mix (Bio-Rad, Hercules, CA, USA), 2.5 mL 1.5MTris-HClbuffer (Tris—Sigma-Aldrich, St. Louis, MO, USA; HCl—Univar, Wilnecote, UK), pH 8.8, 100 μL 10% SDS (Sigma-Aldrich, St. Louis, MO, USA), 4 μL TEMED (Bio-Rad, Hercules, CA, USA), 100 μL 10% APS (Bio-Rad, Hercules, CA, USA); 20 μg venom sample per lane after dissolving in 3 μL of 4× sample loading buffer (Bio-Rad, Hercules, CA, USA) brought up to 12 μL total volume, with DTT (Sigma-Aldrich, St. Louis, MO, USA); reducing conditions were 3 min incubation at 100 °C; gels were run at room temperature for at 120 V (Mini Protean3 power-pack from Bio-Rad, Hercules, CA, USA) for 20 min and then 140 V for 60 min; runs were stopped when dye front was less than 10 mm from the base of the gel. Gels were stained with colloidal coomassie brilliant blue G250 (34% methanol (VWR Chemicals, Tingalpa, QLD, Australia), 3% orthophosphoric acid (Merck, Darmstadt, Germany) 170 g/L ammonium sulfate (Bio-Rad, Hercules, CA, USA), 1 g/L coomassie blue G250 (Bio-Rad, Hercules, CA, USA)) overnight and then destained in 1% acetic acid (Univar, Wilnecote, UK).

### 4.3. Shotgun Sequencing

For shotgun sequencing, reduction and alkylation was performed by dissolving 3 μg of sample in 50 µL of 100 mM ammonium carbonate (Sigma-Aldrich, St. Louis, MO, USA). An amount of 50 μL of 2% iodoethanol/0.5% triethylphosphene (Sigma-Aldrich, St. Louis, MO, USA) in acetonitrile (Scharlau, Barcelona, Spain) was then added to the dissolved samples. The reduced and alkylated sample was resuspended in 20 μL of 40 mM ammonium bicarbonate (Sigma-Aldrich, St. Louis, MO, USA), before being incubated overnight (at 37 °C) with 750 ng sequencing grade trypsin (Sigma-Aldrich, St. Louis, MO, USA). Digestion was stopped by the addition of 1 μL of concentrated formic acid. An amount of 0.75 μg was processed by LC-MS/MS.

### 4.4. LC-MS/MS

LC-MS/MS of both digested gel spots and digested whole venoms (shotgun) was performed using an Agilent (Agilent Technologies, Santa Clara, CA, USA) Zorbax stable bond C18 column (2.1 mm × 100 mm, 1.8 μm particle size, 300 Å pore size) at a flow of 400 μL/min and a gradient of 1%–40% solvent B (90% acetonitrile, 0.1% formic acid) in 0.1% formic acid (Univar, Wilnecote, UK) over 15 min or 4 min for shotgun samples and 2D-gel spots, respectively, on a Shimadzu Nexera UHPLC (Shimadzu, Kyoto, Japan) coupled with an AB SCIEX 5600 Triple TOF mass spectrometer (ABSciex, Mt Waverly, Victoria, Australian). MS^2^ spectra were acquired at a rate of 20 scans/s, with a cycle time of 2.3 s, and optimised for high resolution. Precursor ions were selected between 80 and 1800 *m*/*z*, with a charge state of 2–5, and of an intensity of at least 120 counts per second, with a precursor selection window of 1.5 Da, and excluding isotopes within 2 Da for MS^2^. MS^2^ spectra were searched against known translated cDNA libraries or UniProt databanks with Proteinpilot v4.0 (ABSciex, Mt Waverly, Victoria, Australian) using a thorough identification search, specifying alkylation method (iodoacetamide or iodoethanol), tryptic digestion, and allowing for biological and chemical modifications and amino acid substitions, including artefacts induced by the preparation or analysis processes. This was done to maximize the identification of protein sequences. Spectra were inspected manually to eliminate false positives and only fragments with common artefacts (e.g., Ethanolyl(C) or Deamidated(N)) were retained.

Toxin abbreviations: 3FTx—3-finger toxin; AChE—acetylcholinesterase; ADAM—metalloendopeptidase; CRiSP—cysteine rich secretory protein; CTL—c-type lectin; CYS—cystatin; EN—ectonucleotidase; fXa—activated coagulation factor X homolog; fVa—activated coagulation factor V homolog; HYA—hyaluronidase; KP—kunitz peptide; LAAO—l-amino acid oxidase; NP—natriuretic peptide; NGF—nerve growth factor; PLA_2_—phospholipase A_2_; PLB—phospholipase B; SVMP—snake venom metalloprotease; SVSP—snake venom serine protease; VEGF—vascular endothelial growth factor; VES—vespryn; WP—waprin peptide.

### 4.5. Coagulation Assays

Plasma coagulation assays were carried out using a Hyland-Clotek machine (Hyland, USA). An amount of 20 μL of crude venom at concentrations ranging from 0.39 μg/mL to 200 μg/mL were added to citrated plasma, in the presence and absence of calcium and the time until clot formation measured. Experiments were carried out three times at each concentration point for each calcium condition and the values averaged. Assays in which a clot had not formed after 250 s were stopped. Assays with no result were standardised to 220 s (the longest time after which a clot formed) for graphing. Whole blood coagulation assays were conducted using a Thromboelastograph (TEG-Haemonetics, Braintree, MA, USA). An amount of 320 μL of citrated blood was used, to which 20 μL 0.2 MCaCl was added, to reverse citration. An amount of 20 μL of dilute crude venom (3.125 μg/mL for adult *P. textilis*; 1 mg/mL for neonate *P. textilis*) was added and the results were graphed by the TEG.

## Figures and Tables

**Table 1:** Combined “shotgun” and electrophoresis MS/MS data showing presence and absence of toxin classes detected in the crude venom of 28 species of snake. Toxins marked in red were detected in trace amounts only. See supplementary materials for full details of MS/MS data. (See species abbreviations and toxin abbreviations in materials and methods).

Species	3FTx	AChE	ADAM	CRiSP	CTL	CYS	EN	fXa	fVa	HYA	KP	LAAO	NP	NGF	PLA_2_	PLB	SVMP	SVSP	VEGF	VES	WP
Aan	X	-	-	-	X	-	-	-	-	-	-	X	-	-	X	-	-	-	-	-	-
Amu	X	-	X	X	X	-	-	X	-	-	X	X	-	X	X	-	X	X	-	X	-
Awa	X	-	-	-	-	-	-	-	-	-	-	-	-	-	X	-	-	-	-	X	-
Cbo	X	-	-	-	-	-	-	-	-	X	-	X	-	X	X	-	X	X	X	X	-
Cni	X	-	X	-	-	-	-	X	-	-	-	-	-	X	X	-	X	-	X	-	-
Csq	X	-	-	X	X	-	-	-	-	-	X	X	-	X	X	-	X	-	-	-	-
Dde	X	-	X	X	-	X	-	X	-	X	X	X	-	X	X	X	X	-	X	-	-
Dpa	-	-	-	X	X	-	-	X	-	-	X	X	-	X	X	-	X	-	-	X	-
Dps	-	-	-	X	X	-	-	X	-	X	X	X	-	-	X	-	X	-	X	X	-
Dri	-	-	-	-	X	-	-	X	-	X	X	X	-	-	X	-	X	-	-	X	-
For	X	-	-	-	X	-	-	-	-	-	-	X	-	X	X	-	X	-	-	-	-
Hbi	X	-	-	X	X	-	-	X	-	-	-	X	-	-	X	X	X	-	-	-	-
Hbu	X	-	-	X	-	-	-	X	-	-	X	X	-	-	X	-	X	-	-	-	-
Hst	X	-	-	X	X	-	-	X	-	-	X	X	-	X	X	-	X	-	-	-	-
Hda	X	-	X	X	-	-	X	X	-	X	X	X	-	X	X	X	X	-	X	-	-
Hsi	X	-	X	X	-	-	X	X	-	X	X	X	X	-	X	X	X	-	X	-	-
Lel	X	X	X	X	X	X	-		-	X	X	X	-	X	X	-	X	-	-	X	-
Osc	X	-	-	-	-	-	X	X	X	-	X	-	X	-	X	-	-	-	-	-	-
Omi	X	-	-	-	-	-	X	X	X	-	-	-	X	-	X	-	-	-	-	-	-
Pdw	X	-	-	-	-	-	-	X	-	X	X	-	-	-	X	-	-	X	X	-	-
Psp	X	-	X	X	-	-	-	X	-	X	X	X	-	X	X	-	X	X	X	-	-
Paf(a)	X	-	X	-	-	-	X	X	X		X	-	-	-	X	-	X	-	-	-	-
Paf(b)	X	-	-	-	-	-	-	-	-		X	-	-	-	-	-	-	-	-	-	-
Pte(a)	X	-	-	X	-	-	-	X	X		X	-	-	-	X	-	-	-	-	-	-
Pte(b)	X	-	-	X	-	-	-	-	-	-	X	-	-	-	X	-	-	-	-	-	-
Sbe	X	-	-	X	X	-	-	-	-	-	-	-	-	X	X	-	X	-	-	-	-
Sfa	X	-	X	X	-	-	X	X	-	X	X	-	-	X	X	-	X	-	X	-	-
Spu	X	-	X	X	-	-	-	X	-	-	X	X	X	-	X	-	X	-	X	-	-
Ssu	X	-	X	X	-	-	-	-	-	-	-	-	-	-	X	-	X	-	X	-	-
Van	X	-	-	X	-	X	-	-	-	-	X	-	-	X	-	X	X	-	-	-	X

## Supplementary Materials

The following are available online at www.mdpi.com/2072-6651/8/11/309/s1.
